# Differences in decisions affected by cognitive biases: examining human values, need for cognition, and numeracy

**DOI:** 10.1186/s41155-023-00265-z

**Published:** 2023-09-07

**Authors:** Regis K. Kakinohana, Ronaldo Pilati

**Affiliations:** Institute of Psychology, University of Brasilia, Brasilia, DF 72910-000 Brazil

**Keywords:** Biases, Decisions, Human values, Individual differences, Need for cognition, Numeracy

## Abstract

A better understanding of factors that can affect preferences and choices may contribute to more accurate decision-making. Several studies have investigated the effects of cognitive biases on decision-making and their relationship with cognitive abilities and thinking dispositions. While studies on behaviour, attitude, personality, and health worries have examined their relationship with human values, research on cognitive bias has not investigated its relationship to individual differences in human values. The purpose of this study was to explore individual differences in biased choices, examining the relationships of the human values self-direction, conformity, power, and universalism with the anchoring effect, the framing effect, the certainty effect, and the outcome bias, as well as the mediation of need for cognition and the moderation of numeracy in these relationships. We measured individual differences and within-participant effects with an online questionnaire completed by 409 Brazilian participants, with an age range from 18 to 80 years, 56.7% female, and 43.3% male. The cognitive biases studied consistently influenced choices and preferences. However, the biases showed distinct relationships with the individual differences investigated, indicating the involvement of diverse psychological mechanisms. For example, people who value more self-direction were less affected only by anchoring. Hence, people more susceptible to one bias were not similarly susceptible to another. This can help in research on how to weaken or strengthen cognitive biases and heuristics.

Daily choices are made about strategies and investments in Education, Health, Science, Public Safety, and the Economy. A better understanding of the factors that can affect preferences and choices contributes to more accurate decision-making. Many studies have investigated the effects of cognitive biases on decision-making. For example, in the study conducted by Quattrone and Tversky (1988), when participants had to choose between an economic program J, in which 10% of people would be unemployed with an inflation rate of 12%, and an economic program K, in which 5% of people would be unemployed with an inflation rate of 17%, 64% of participants preferred program K. When participants had to choose between an economic program J, in which 90% of people would be employed with an inflation rate of 12%, and an economic program K, in which 95% of people would be employed with an inflation rate of 17%, 54% of participants preferred program J. Although the two situations were the same, the choices were biased by minor changes in the way the program results were presented (Kahneman & Tversky, 1984; Tversky & Kahneman, 1981).

Some researchers explored the relationship between these biases with cognitive abilities and thinking dispositions (e.g., Šrol & De Neys, 2021; Toplak & Flora, 2021; Toplak et al., 2014; West et al., 2008; Wyszynski & Diederich, 2023). At the same time, many studies on behavior, such as political behavior; on prediction of attitudinal variables, such as ethical dilemmas; on relationships with personality traits, such as conscientiousness; and health worries, such as COVID-related worries, have used human values in their investigations (e.g., Bojanowska & Urbańska, 2021; Fischer et al., 2021; Goren et al., 2022; Grosz et al., 2021; Klein & Ben Hador, 2021; Mubako et al., 2021). However, we found no research investigating whether individual differences in human values are related to cognitive biases. Could it be that people who prioritize self-direction are less affected by cognitive biases? And are people who value conformity more affected? Therefore, our study involved two topics, cognitive biases and human values, widely studied separately, but not together. This work investigated the relationships between human values and decisions affected by cognitive biases. In addition, we analysed whether these relationships were mediated by the need for cognition and moderated by numeracy.

## Cognitive biases

Research on decision-making is voluminous and multidisciplinary, being investigated in several areas of Psychology, Economics, and Sociology. Many of these studies involve the discussion between the normative approach, which focuses on rationality and expected logic in choices, and the descriptive approach, which deals with people’s beliefs and preferences in the real world (Kahneman & Tversky, 1984). In normative models, the decision maker is rational and has a stable system of preferences, as well as the knowledge and skills necessary to choose the best available alternative. In descriptive models, decision-makers do not have enough cognitive capacity or time to always analyse all possible alternatives, often not using the processing mechanisms that require greater mental effort (Kahneman, 2011; Simon, 1955; Stanovich, 2018; Thaler & Sunstein, 2009). Since the 1970s, theories about the dual model of cognitive processes have been discussed in research on decision-making (Evans & Stanovich, 2013; Kahneman, 2011). Type 1 processes are intuitive, fast, and automatic and do not require a great deal of mental effort, while type 2 processes are thoughtful, slow, controlled, and require more cognitive effort (Evans & Stanovich, 2013; Kahneman, 2011; Stanovich, 2016, 2018). Heuristics are mental shortcuts that replace complex questions with simpler ones. Through heuristics, type 1 processes generate quick answers to difficult problems. Type 2 processes may reject a heuristic answer or modify it, but often simply accept the answer without expending effort on evaluating it (Kahneman, 2011). Heuristic answers are not necessarily wrong. On the contrary, in most cases, they are good enough and more appropriate when there is neither time nor data for an exhaustive diagnosis (Marewski & Gigerenzer, 2012; Mousavi & Gigerenzer, 2017). However, in situations that require and allow more detailed analysis, such as decisions in administration, investments, and legal judgments, type 2 processes must be more committed to monitoring, endorsing, rejecting, or modifying these responses (Berthet, 2022; Bystranowski et al., 2021; Kahneman, 2011; Neal et al., 2022; Stanovich, 2016). Cognitive biases are systematic errors people make in choices and estimates when type 2 processes rely on heuristic responses and fail to detect their divergence from logical, mathematical, and statistical foundations (Kahneman, 2011; Tversky & Kahneman, 1974).

Some examples of cognitive biases are (a) anchoring effect, (b) framing effect, (c) certainty effect, and (d) outcome bias. The anchoring effect occurs when insufficient adjustments influence estimates concerning these initial values (Epley & Gilovich, 2001; Kahneman, 2011; Tversky & Kahneman, 1974). Tversky and Kahneman (1974) found that participants estimated a higher percentage of African countries at the United Nations after considering a higher anchor than a lower anchor. Before participants made their estimates, the researchers spun a lucky wheel. When the wheel stopped at 10 the average estimate was that 25% of the countries in the United Nations were from Africa, while when the wheel stopped at 65 the estimate increased to 45% (Kahneman, 2011; Tversky & Kahneman, 1974). The framing effect occurs when choices are affected by small changes in presentation form (Kahneman & Tversky, 1984; Quattrone & Tversky, 1988; Tversky & Kahneman, 1981). Roberts and Wernstedt (2019) found that emergency managers in the USA changed their preference between two action plans presented according to the presentation format, in terms of homes saved or homes destroyed. When participants had to choose between a plan that would result in the destruction of 75 of 100 homes at risk and another that had a 75% chance of destroying all 100 homes and a 25% chance of destroying no home, 89% of these participants preferred the latter plan. When participants had to choose between a plan that would result in saving 25 of 100 homes and another that had a 25% chance of saving all 100 homes and a 75% chance of saving no homes, 58% of these participants preferred the former plan. The certainty effect occurs when the same reduction in probability has a greater impact if an event is considered certain and not only probable (Tversky & Kahneman, 1981). In the study of Tversky and Kahneman (1981), when participants had to choose between a sure win of $30 versus an 80% chance to win $45, 78% of these participants preferred the former option, and when participants had to choose between a 25% chance to win of $30 versus a 20% chance to win $45, 58% of these participants preferred the latter option. Outcome bias occurs when the assessment of the quality of a decision is influenced by its outcome, regardless of the information available at the time it was made (Baron & Hershey, 1988). Baron and Hershey (1988) presented participants with a common decision about the surgical procedure. Some participants were told that the operation was a success, while others that it was a failure. Participants evaluated the decision more positively when the outcome was a success than when the outcome was a failure.

Šrol and De Neys (2021) explored cognitive skills, readiness for analytical thinking, numeracy, cognitive reflection, and knowledge of logical principles as predictors of individual differences in detecting biases, identifying knowledge of logical principles as the best predictor of these differences. Toplak et al. (2014) examined the relationship between performance on tasks involving cognitive biases, such as resistance to the framing effect, with individual differences related to cognitive abilities and thinking dispositions, identifying only verbal and non-verbal intelligence as a significant predictor of resistance to framing. West et al. (2008) investigated the relationship between cognitive ability and thinking dispositions with performance in problems involving heuristics and biases, such as the framing effect, finding positive associations. Studies use terms such as susceptibility and resistance to refer to the size of the bias being greater and smaller, respectively, to facilitate the understanding of the results (e.g., Šrol & De Neys, 2021; Toplak & Flora, 2021; Toplak et al., 2014). For instance, greater resistance to anchoring refers to a smaller anchoring effect. All these works did not explore human values. Research on the relationships of individual differences with heuristics and biases has focused on cognitive abilities and thinking dispositions (Berthet & De Gardelle, 2023).

## Human values

**Fig. 1:**
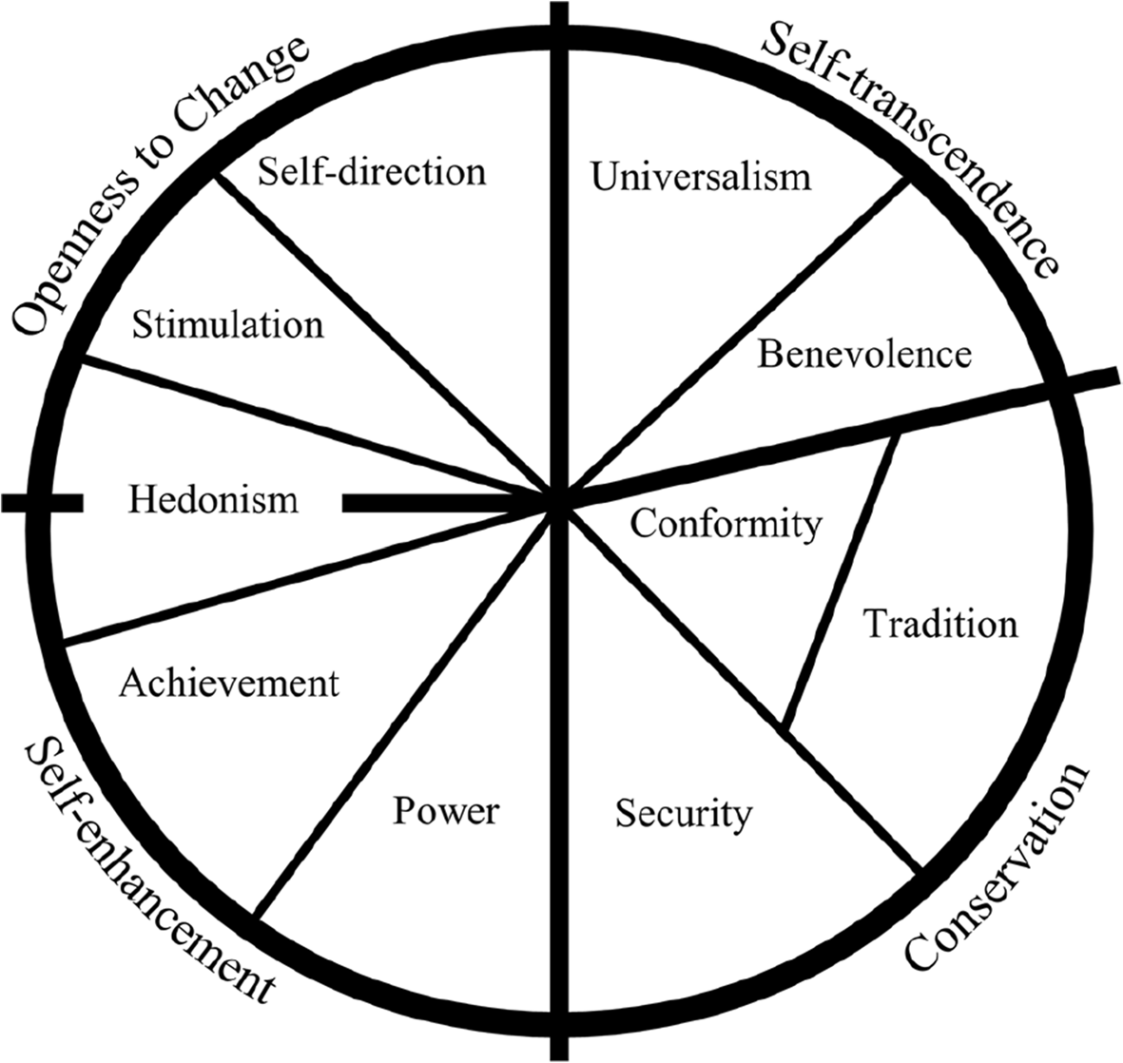
Theoretical circular structure of ten human values (Schwartz, 1992, 2012)

Research has investigated human values relations with personality traits, creativity, subjective well-being, religiosity, prejudice, and prosocial, political, and environmental behaviour (Sagiv & Schwartz, 2022). Despite this, cognitive bias research has not explored individual differences in human values, to the best of our knowledge. Search in APA PsycNet, Web of Science, and PubMed, combining the keywords “cognitive bias,” “individual differences,” and “human values,” found no matches. We performed this search on August 7, 2023, without any other restriction criteria. Human values are concepts and beliefs, which lead to desirable states or behaviors, guiding the selection and evaluation of behaviors and events. They transcend specific situations and are ordered according to relative importance, acting as motivators of decision-making, attitudes, and individual behaviors (Schwartz, 1992, 2006a, 2012). The Theory of Basic Human Values identifies and defines ten values in a circular structure: (a) self-direction, (b) stimulation, (c) hedonism, (d) achievement, (e) power, (f) security, (g) conformity, (h) tradition, (i) benevolence, and (j) universalism, as shown in Fig. 1 (Sagiv & Schwartz, 2022; Schwartz, 1992, 2012). Subsequently, the theory was refined, distinguishing nineteen values that remained in correspondence with the original ten values (Sagiv & Schwartz, 2022; Schwartz, 2016; Schwartz & Cieciuch, 2022; Schwartz et al., 2012). The circular structure represents the relations between the values, where actions in search of any value have consequences that conflict with some values but are congruent with others, illustrating how these values are organized into two polar dimensions: openness to change—conservation, which captures conflict between values that emphasize independence of thought and readiness for change and values that emphasize self-control and resistance to change, and self-transcendence—self-enhancement, which captures conflict between values that emphasize concern for others and values that emphasize dominance over others (Schwartz, 2012). It is expected that if a value on one side of the circular structure induces certain behaviour, values on the opposing side should inhibit that behaviour (Schwartz, 2017). In the openness to change—conservation dimension, self-direction is a value related to independent and autonomous thinking, while conformity, in the opposite direction, is related to restricting actions that may upset others and violate social expectations. In the self-transcendence—self-enhancement dimension, universalism is related to the concern for the understanding and well-being of all people, while power, in the opposite direction, is related to control over people and resources (Schwartz, 1992).

From the individual level of the Theory of Basic Human Values, Schwartz (2006b) developed the Theory of Cultural Value Orientations, which defines seven cultural value orientations that characterize societies based on the value priorities of individuals in each society: (a) intellectual autonomy, (b) affective autonomy, (c) embeddedness, (d) egalitarianism, (e) hierarchy, (f) harmony, and (g) mastery. These cultural value orientations consider values prevalent among people in a society that express shared notions of what is good and desirable in that culture, influencing individual beliefs, actions, and goals (Schwartz, 2006b). The study of Kakinohana et al. (2023) found that cultural value orientations explained substantial amounts of variability in anchoring effect sizes between cultures. At the societal level, intellectual autonomy, harmony, and egalitarianism cultural value orientations were negatively correlated with anchoring effect size (Kakinohana et al., 2023). However, we found no research on the relationship between cognitive biases and individual human values, let alone relating individual human values to multiple biases in the same research design. Therefore, this study investigated the relationship between human values and four cognitive biases at the individual level.

The defining aim of self-direction is independent thought, and it is associated with freedom, creativity, and curiosity, as the intellectual autonomy cultural value orientation (Kharlamov & Pogrebna, 2021; Schwartz, 1992, 2006b, 2012). Intellectual autonomy, which encourages individuals to pursue their own ideas and intellectual directions (Schwartz, 2006b), presents a negative correlation with anchoring effect size (Kakinohana et al., 2023). In the opposite direction of self-direction, conformity emphasizes self-restraint in interactions (Schwartz, 1992, 2012). To explore whether concerns with independent thought were negatively associated with the bias effects, while self-restriction was positively associated, we investigated whether people who value self-direction more would be less affected by cognitive biases and whether people who value conformity more would be more affected. The defining goal of hedonism is pleasure (Schwartz, 1992, 2012). Since hedonism and self-direction are adjacent values and the division between them is arbitrary, there is often no difference between them in real data (Tamayo & Porto, 2009). Therefore, we decided not to explore hedonism in this study.

The defining aim of universalism is to understand and protect the well-being of all people and nature (Schwartz, 1992, 2012). It is associated with a world at peace and a world of beauty, as harmony, and with equality and social justice, as egalitarianism (Kharlamov & Pogrebna, 2021; Schwartz, 1992, 2006b, 2012). At the cultural level, harmony, which emphasizes trying to understand and appreciate the world rather than exploit it, and egalitarianism, which seeks to induce people to recognize one another as equals (Schwartz, 2006b), present negative correlations with anchoring effect size (Kakinohana et al., 2023). In the opposite direction of universalism, power emphasizes dominance over people and resources (Schwartz, 1992, 2012). To explore whether concerns with understanding and treating people as equals were negatively associated with the bias effects, while dominance over people and resources were positively associated, we investigated whether people who value universalism more would be less affected by cognitive biases and whether people who value power more would be more affected.

The defining aim of benevolence is to protect the well-being of close others (Schwartz, 1992, 2012). Since benevolence and universalism are adjacent values and the division between them is arbitrary, there is often no difference between them in real data (Tamayo & Porto, 2009). Consequently, we decided not to explore benevolence in this study. The defining goal of achievement is personal success through a demonstration of competence (Schwartz, 1992, 2012). Since achievement and power are adjacent values and the division between them is arbitrary, there is often no difference between them in real data (Tamayo & Porto, 2009). Therefore, we decided not to explore achievement in this study. The defining aim of stimulation is excitement, and it is associated with an exciting life, as affective autonomy (Kharlamov & Pogrebna, 2021; Schwartz, 1992, 2006b, 2012). At the cultural level, affective autonomy, which encourages individuals to pursue affectively positive experiences (Schwartz, 2006b), did not present correlations with three of the four anchoring effect sizes studied by Kakinohana et al. (2023). Consequently, we decided not to explore stimulation in this study. Tradition, related to respect for religious and cultural customs, and security, related to safety, are associated with devoutness and national security, as the cultural value orientation embeddedness (Kharlamov & Pogrebna, 2021; Schwartz, 1992, 2006b, 2012). Embeddedness, which emphasizes maintaining the traditional order (Schwartz, 2006b), did not present correlations with two of the four anchoring effect sizes studied by Kakinohana et al. (2023). Therefore, we decided not to explore tradition and security in this study.

## Need for cognition and numeracy

The need for cognition represents the tendency of people to engage in and enjoy tasks that require cognitive effort (Cacioppo & Petty, 1982). Individuals with a higher need for cognition tend to seek and reflect more on information to adopt positions and solve problems, while individuals with a lower need for cognition are more likely to use cognitive heuristics (Cacioppo et al., 1996). Several studies have used the need for cognition as a measure of readiness for analytical thinking, as well as some research has investigated the relationship between the need for cognition, personality factors, intelligence, attitudes, and behaviors (e.g., Cacioppo et al., 1996; Fleischhauer et al., 2010; Furnham & Thorne, 2013). The previously mentioned studies by Šrol and De Neys (2021), by Toplak et al. (2014), and by West et al. (2008) used the need for cognition as a measure of thinking disposition. Furthermore, Thomson and Oppenheimer (2016) identified that the need for cognition was positively related to cognitive reflection and resistance to belief bias, and Carnevale et al. (2011) investigated the relationship between the need for cognition and some decision-making skills, observing that participants with a greater need for cognition were more resistant to the framing effect. However, investigation of the relationship between the need for cognition and the framing effect has shown mixed results. While some studies indicated that the need for cognition moderates framing effects, others did not identify a significant relation (Wyszynski & Diederich, 2023).

As human values act as motivators of attitudes and behaviors, they also influence the need for cognition. People who value more self-direction, a human value that motivates independent thoughts and actions, tend to have a higher need for cognition, while those who value more conformity, a human value that motivates the preservation of the status quo, tend to have a lower need for cognition (Coelho et al., 2020). Thus, in addition to the direct route between human values and cognitive biases, we also investigated the indirect route through the need for cognition mediation.

To be able to identify divergences with heuristic responses, type 2 processes need an apparatus of logical, mathematical, and statistical skills, even if this apparatus is not sufficient to avoid their endorsement (Kahneman, 2011; Stanovich, 2018). However, the role of the fundamental skills apparatus for good performance in tasks with biases and heuristics is still little explored (Stanovich, 2018). Numeracy is the ability to process mathematical concepts and basic probability, and it is more than just mathematical skills, involving these skills’ practical applications and the reasoning associated (Lipkus et al., 2001; Reyna & Brainerd, 2023). Some studies have investigated the relationship of numeracy with susceptibility to bias (Reyna & Brainerd, 2023). These works investigated both objective numeracy, through objective questions with numerical answers that measure the ability to understand information in numerical format, and subjective numeracy, through self-reported items related to the ability to use numerical information and the preference for presenting information in numerical format (Fagerlin et al., 2007; Lipkus et al., 2001; Schwartz, 1997).

Peters et al. (2006) indicated that participants with lower numeracy had a greater framing effect. Ghazal et al. (2014) identified that statistical numeracy, related to understanding statistical and probabilistic calculations, such as comparing and transforming probabilities and proportions (Cokely et al., 2012), was a robust predictor of better performance in problems related to denominator neglect, which occurs when the individual evaluates a probability only by the numerator, disregarding the denominator (Kahneman, 2011; Yamagishi, 1997). Since many bias tasks involve an understanding of percentages and probabilities, numeracy is necessary to perform well in these heuristics and bias tasks (Šrol & De Neys, 2021; Stanovich, 2018; Stanovich & West, 2008; Toplak et al., 2014). Thus, even if people who value self-direction more, for example, are encouraged to think independently and autonomously, if the task requires numeracy skills, which are not present, they will tend to trust the heuristic response, as normative declarative knowledge required will not be available to verify it. Therefore, we explored the possibility of numeracy acting as a moderator of the relationship between human values and the need for cognition with cognitive biases.

## The present study

This study aimed to investigate the relationships between human values and the following cognitive biases: (a) anchoring effect, (b) framing effect, (c) certainty effect, and (d) outcome bias. We selected these four biases, expecting that different biases would have similar relationships with human values. The study also intended to analyse the effects of the mediation of the need for cognition and the moderation of numeracy in these relationships. Given the absence of previous evidence between individual human values and cognitive biases, we explored whether self-direction would have a negative relationship with the effects of all four studied biases, whether universalism would have a negative relationship with these effects, whether conformity would have a positive relationship with these effects, whether power would have a positive relationship with these effects, whether the need for cognition would mediate the relationships between the studied human values and these effects, and whether numeracy would moderate these relationships. This study was approved by the Research Ethics Committee in Human and Social Sciences of the University of Brasília. We pre-registered this project to the Open Science Framework on June 24, 2021, before data collection (OSF; https://osf.io/jzncd). We updated this registration, on December 14, 2021, to provide transparency about adjustments needed to address issues identified only after data collection. The original pre-registration is still available for access.

## Method

### Participants

The sample consisted of 409 Brazilian adults. Their age ranged from 18 to 80 years (*M* = 40.5, *SD* = 14.3), with 56.7% female and 43.3% male. We estimated that we would need 725 participants, using the G*Power 3.1.9.6 software and assuming a small effect size (*f*^2^ = .02), *α* = .05, and *β* = .20 (Cohen, 1988; Faul et al., 2009; Field, 2018). The invitation to participate was through emails and social networks Facebook, Twitter, Instagram, and LinkedIn. Within the deadline established for data collection, from June 25, 2021, to August 25, 2021, 448 participants completed the online questionnaire. The initial page of the questionnaire presented the research information on the Informed Consent Form, requiring the participant to give written consent by ticking a checkbox before proceeding. Participation was anonymous, with no possibility of identifying individual responses. We excluded 32 participants who failed the attention items and 7 participants identified as outliers by the Mahalanobis distance with* p* < .001 (Tabachnick & Fidell, 2018), resulting in a sample of 409 Brazilian adults. We defined these exclusion criteria in the pre-registration. Although we did not reach the estimated size, also using the G*Power 3.1.9.6 software, the sensitivity calculation indicated a required effect size between small and medium (*f*^2^ = .04).

### Measures

#### Cognitive biases

In the experimental part, we used problems with choices associated with the studied cognitive biases: (a) anchoring effect, (b) framing effect, (c) certainty effect, and (d) outcome bias. Each problem had two or four versions. The differences between versions varied according to the strategy to measure the studied bias and to reduce the risk of perception of the investigated pattern in a within-participant design. These instruments are available on OSF (https://osf.io/twqsu). In the pre-registration (https://osf.io/jzncd), we also described between-participants measures that would be used in case of major problems with within-participant design.

We used two questions based on the study carried out by Tversky and Kahneman (1974) to measure the anchoring effect. Each participant responded to the two versions in random order. The low anchor version informed that approximately 10 thousand people lived in the city of Itabi—Sergipe (Brazilian state) and in the city of Nova Erechim—Santa Catarina (Brazilian state) and asked how many thousand people approximately the respondent thought to live in the city of Poço das Trincheiras—Alagoas (Brazilian state) and in the city of Sobradinho—Rio Grande do Sul (Brazilian state), while the high anchor version informed that approximately 90 thousand people lived in the city of Itabaianinha—Sergipe and in the city of Laguna—Santa Catarina and asked the same question. The answers were in open numerical format. We calculated the within-participant anchoring effect by the difference between the estimates of each participant, considering the first estimate as the self-generated anchor (Epley & Gilovich, 2001). That is, we considered that the response to the second stimulus would be more influenced by the response provided to the first stimulus than by the information presented in the second stimulus. Thus, smaller differences between the two answers given by the participant indicate greater anchoring. As larger values represented smaller effects, to facilitate the understanding of the results, we used the term anchoring resistance in the examination of the relationships. Less resistance to anchoring refers to smaller differences between the second stimulus and the first one that acted as an anchor. During the analysis of the responses, we identified a problem in the anchoring responses. Although the statement asked for the value in thousands of people and we added the text “thousand people” to the side of the answer field, it is possible that some participants considered the total value in their answers. For example, the biggest answer was 200,000 thousand people, that is, 200 million people, a number close to the total Brazilian population. Due to the uncertainty regarding these answers, values equal to or greater than 1000 thousand people, that is, one million people, were not considered in the analyses involving the anchoring effect. Therefore, we disregarded 46 responses in the anchoring-related analyses. Even with these exclusions, the sensitivity calculation indicated a required effect size between small and medium (*f*^2^ = .04).

We investigated the framing effect through four questions based on the work of Larrick and Soll (2008), which showed the same consumption information and car exchange options, but with different presentation formats. We presented the information in km/l and km/m^3^ in the framed versions, while in litres per 100 km and m^3^ per 100 km in the unframed versions. Responses were on a seven-point scale from (1) *definitely changing car “A”* to (7) *definitely changing car “B”*. Changing car “A” was the normative choice. Each participant randomly answered one framed and one unframed question, one using liter and the other using m^3^. For example, one participant answered a question that presented consumption information in km/l and a question that presented consumption information in m^3^ per 100 km. We measured the within-participant framing effect by the difference between responses to the framed and unframed versions of each participant. Greater differences indicate a greater preference for non-normative choice and, therefore, a greater framing effect.

To measure the certainty effect, we used two questions based on the study carried out by Tversky and Kahneman (1981). One question offered a sure win of BRL 30.00 or an 80% chance of winning BRL 45.00, while the other offered a 25% chance of winning BRL 30.00 or a 20% chance of winning BRL 45.00. Responses were on a seven-point scale from (1) *I definitely prefer option A* to (7) *I definitely prefer option B*. Each participant answered both versions in random order. We calculated the within-participant certainty effect by the difference between the certain gain version response and the response to the version without the certain gain option. Larger differences indicate a larger effect.

We used four questions based on the study performed by Baron and Hershey (1988) to measure outcome bias. The questions either presented a positive or negative result for a decision made by a mayor or by an investor. Responses were on a seven-point scale from (1) *clearly incorrect* to (7) *clearly correct*. Each participant randomly responded to one positive and one negative version, one from the mayor and the other from the investor. For example, the questionnaire presented a positive result for a decision made by a mayor and a negative result for a decision made by an investor for one participant to evaluate. We measured the within-participant outcome bias effect by the difference between responses to the positive and negative versions of each participant. Greater differences indicate a greater effect.

#### Human values

We measured human values with 21 of the 57 items from the Revised Portrait Values Questionnaire (PVQ-RR; Schwartz & Cieciuch, 2022), adapted for Brazil by Torres et al. (2016). The PVQ-RR has female and male versions. The female version uses “How much does this person look like you?”, while the male version uses “How much does this guy look like you?” An example of a self-direction thought item in the female version is “It is important to her to form her views independently”, and an example of a universalism-concern item in the male version is “It is important to him that the weak and vulnerable in society be protected”. Responses were on a six-point scale from (1) *not at all like me* to (6) *very much like me*. We used the three self-direction action items and the three self-direction thought items to measure self-direction (*α* = .78), the three conformity-rule items and the three conformity-interpersonal items to measure conformity (*α* = .77), the three universalism-tolerance items and the three universalism-concern items to measure universalism (*α* = .72), and the three power resources items to measure power (*α* = .77).

#### Need for cognition

We measured the need for cognition with a Brazilian ten-item version of the Need for Cognition Scale (*α* = .84; Cacioppo et al., 1984; Caldas et al., 2019). Responses were on a five-point scale from (1) *not at all characteristic* to (5) *totally characteristic*. This version is available on OSF (https://osf.io/twqsu).

#### Numeracy

We measured numeracy with two instruments. The General Numeracy Scale (Lipkus et al., 2001; Schwartz, 1997) is composed of three objective questions (*α* = .66) and measures the ability to understand information in numerical format, basic probability, and mathematical concepts (Liberali et al., 2012). The Subjective Numeracy Scale (Fagerlin et al., 2007) is composed of eight items (*α* = .86) related to the ability to use numerical information and the preference for presenting information in numerical format, which was answered on a six-point scale (Liberali et al., 2012).

#### Procedure and data analysis

We made a questionnaire available on the Internet through the Enterprise Feedback Suite (EFS; https://ww3.unipark.de/www/front.php). The EFS is a platform that allows the creation of web surveys with various question types. Responses are collected through the questionnaire made available on its website and data are accessed by a secure login system. Before responding, the participants gave their informed consent. The questionnaire flowchart, including the stimuli randomization strategy and the order of presentation, is available in the supplemental material on OSF (https://osf.io/twqsu).

For the mediation analyses, we used the macro PROCESS v3.5 (Hayes, 2018), with the robust option of violating HC3 (Heteroscedasticity Consistent 3; Long & Ervin, 2000). We performed the statistical analyses in IBM (International Business Machines) SPSS (Statistical Package for the Social Sciences) Statistics (version 27). The syntaxes and outputs are also available on OSF (https://osf.io/twqsu). We also provided a file with unidentified data and an R script, which makes it possible to reproduce almost all the SPSS results.

## Results

### Anchoring effect

**Fig. 2:**
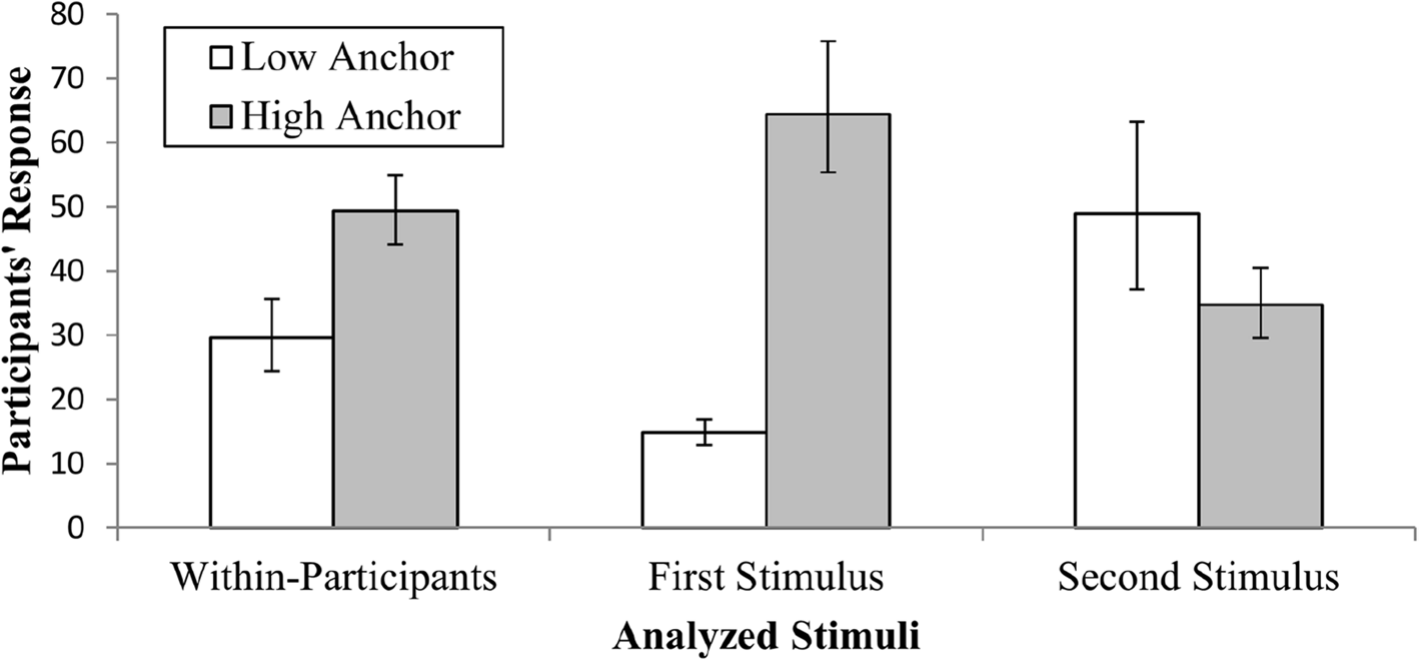
Participant’s responses to anchoring stimuli. *Note. N* = 363. Error bars show 95% bias-corrected accelerated bootstrap confidence intervals

In the within-participant analysis, participants estimated a lower value (*M* = 29.6, *SD* = 55.7) when they received the low-anchor stimulus than when they received the high-anchor stimulus (*M* = 49.3, *SD* = 56.8). This difference of 19.7, 95% CI [13.4, 26.1], was significant, *t*(362) = 6.13, *p* < .001, representing an effect *d* = 0.32, 95% CI [0.22, 0.43]. Figure 2 shows that, in the first stimulus, participants estimated a lower value for the low-anchor (*M* = 14.9, *SD* = 15.4) and a higher value for the high-anchor (*M* = 64.4; *SD* = 69.0), *d* = 1.00, 95% CI [0.78, 1.21]. In the second stimulus, they estimated a higher value in the low-anchor (*M* = 49.0, *SD* = 95.6) than in the high-anchor (*M* = 34.7, *SD* = 34.9), *d* = 0.20, 95% CI [0.00, 0.41].

In the analysis of the resistance to anchoring, *F*(5, 357) = 3.25, *p* = .007, *R*^2^ = .04, Table 1, we identified a statistically significant positive direct effect of self-direction, which means that the greater the self-direction, the lower the anchoring effect. Its total effect was also statistically significant, 8.30, 95% CI [1.97, 14.63], *p* = .010, as well as its indirect effect mediated by need for cognition, −2.05, 95% CI [−4.41, −0.48], which means that the greater the need for cognition, the greater the anchoring. We did not identify other statistically significant direct effects, indirect effects, and interactions.

**Table 1:** Mediation model of the resistance to anchoring

Variable	*B*	*SE*	95% CI for *B*	*p*
Self-direction	10.35	3.50	[3.47, 17.24]	.003
Conformity	0.98	2.10	[−3.16, 5.12]	.641
Power	1.42	2.72	[−3.92, 6.77]	.601
Universalism	−4.73	2.51	[−9.67, 0.20]	.060
Constant	26.62	3.05	[20,63, 32,61]	<.001
Need for cognition	−8.06	3.39	[−14,72, −1,40]	.018

*Note. N* = 363. *B* coefficients generated with model 4 of the PROCESS macro; *CI* confidence interval

### Framing effect

**Fig. 3:**
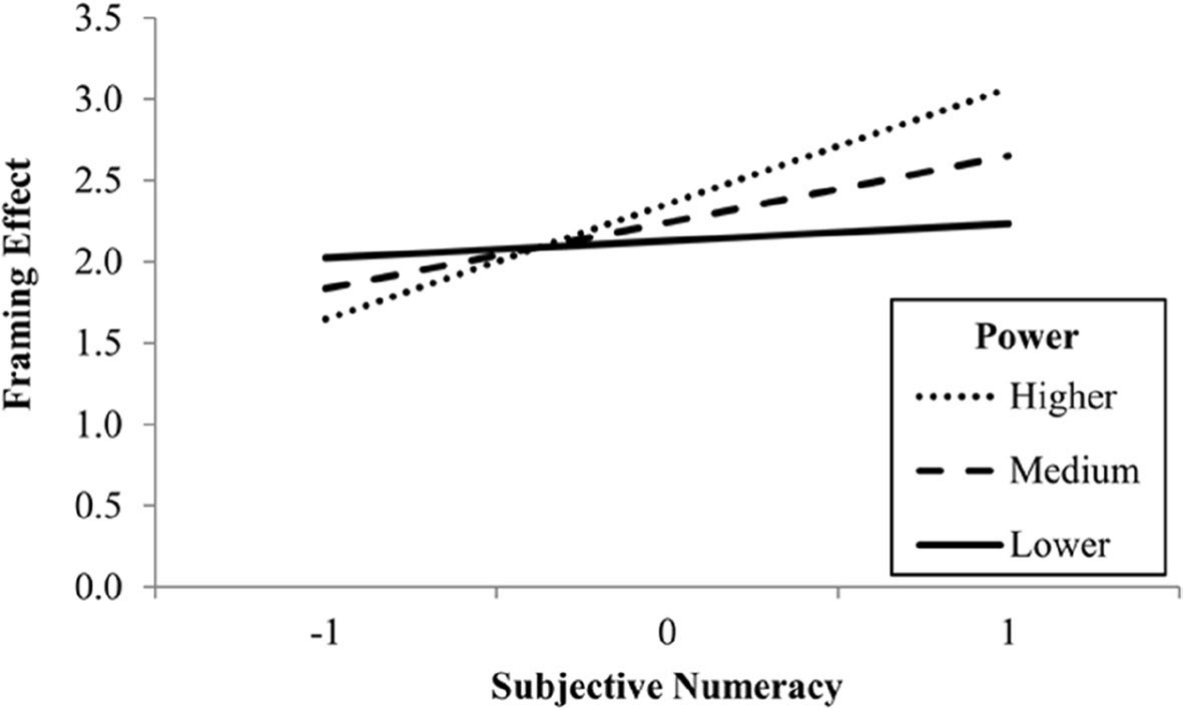
Moderation of the effect of power on framing effect by subjective numeracy. *Note.* Subjective numeracy and power values are standardized. Higher, medium, and lower lines represent mean plus one *SD*, mean, and mean minus one *SD*, respectively

**Fig. 4:**
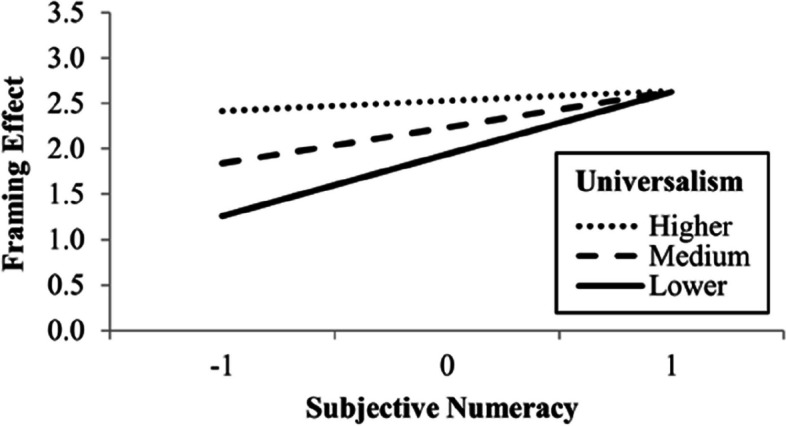
Moderation of the effect of universalism on framing effect by subjective numeracy. *Note.* Subjective numeracy and universalism values are standardized. Higher, medium, and lower lines represent mean plus one *SD*, mean, and mean minus one *SD*, respectively

Participants preferred to change car “B”, the non-normative choice, more in the framed versions (*M* = 4.93, *SD* = 2.28) than in the unframed versions (*M* = 2.67, *SD* = 2.03). This difference of 2.27, 95% CI [1.98, 2.56], was significant, *t*(408) = 15.36, *p* < .001, *d* = 0.76, 95% CI [0.65, 0.87]. We conducted a one-way ANOVA (analysis of variance) before grouping the two framed and the two unframed versions. This analysis is in the supplemental material. The framing effect model, *F*(5, 403) = 1.45, *p* = .207, *R*^2^ = .02, did not indicate any statistically significant direct and indirect effects. However, conditional process analyses indicated statistically significant interactions of subjective numeracy with power, *F*(1, 401) = 4.06, *p* = .045, Δ*R*^2^ = .01, and with universalism, *F*(1, 401) = 7.07, *p* = .008, Δ*R*^2^ = .01. Figure 3 shows that when they presented higher subjective numeracy, participants who valued power more had a greater framing effect, while Fig. 4 shows that when they presented lower subjective numeracy, participants who valued universalism less had a smaller framing effect.

### Certainty effect

**Fig. 5:**
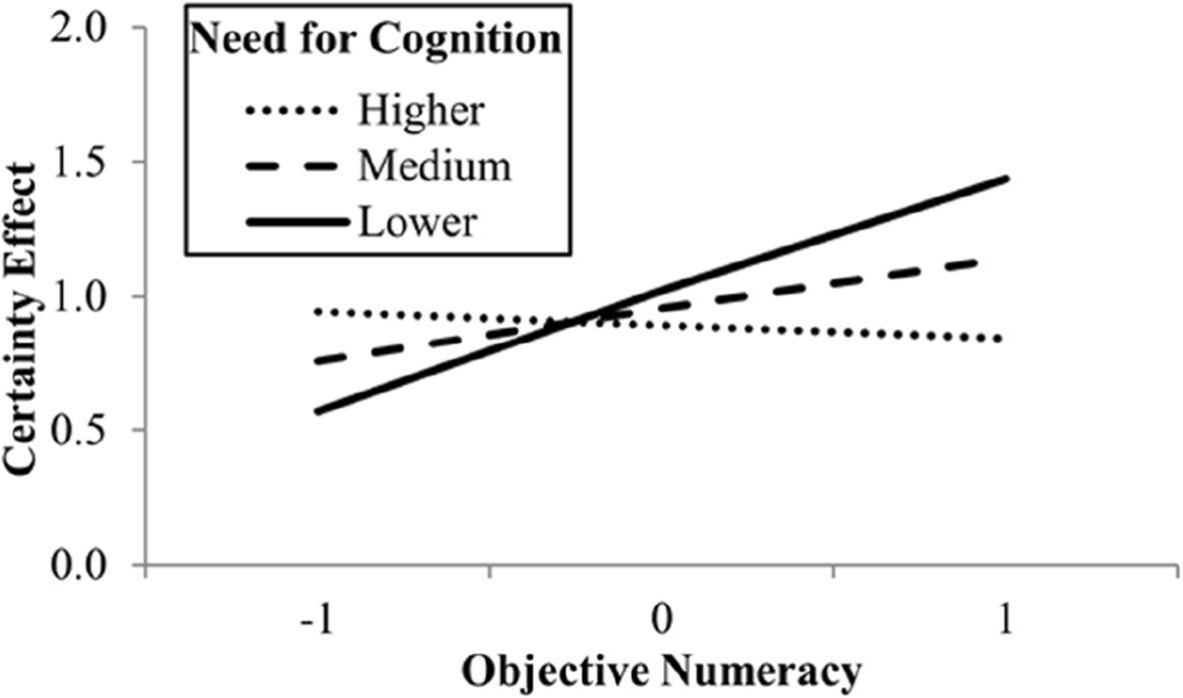
Moderation of the effect of need for cognition on certainty effect by objective numeracy. *Note.* Objective numeracy and need for cognition values are standardized. Higher, medium, and lower lines represent mean plus one *SD*, mean, and mean minus one *SD*, respectively

Participants preferred the least likely option more when there was no certain gain alternative (*M* = 3.94, *SD* = 2.11) than when there was such an alternative (*M* = 3.05, *SD* = 2.04). This difference of 0.89, 95% CI [0.64, 1.13], was significant, *t*(408) = 7.07, *p* < .001, *d* = 0.35, 95% CI [0.25, 0.45]. The certainty effect model, *F*(5, 403) = 1.62, *p* = 0.15, *R*^2^ = .02, shown in Table 2, indicated that only power had a statistically significant negative direct effect, which means that the greater the power, the smaller the certainty effect. No interaction with subjective numeracy was statistically significant, but objective numeracy interacted with the need for cognition,* F*(1, 401) = 4.02, *p* = .046, Δ*R*^2^ = .01. Figure 5 shows that when they presented higher objective numeracy, participants with lower need for cognition had greater certainty effect.

**Table 2:** Mediation model of the certainty effect

Variable	*B*	*SE*	95% CI for *B*	*p*
Self-direction	0.21	0.13	[−0.03, 0.46]	.091
Conformity	0.02	0.14	[−0.25, 0.29]	.871
Power	−0.31	0.13	[−0.57, -0.05]	.018
Universalism	−0.13	0.15	[−0.43, 0.16]	.371
Constant	0.89	0.13	[0.64, 1.13]	<.001
Need for cognition	0.00	0.13	[−0.26, 0.26]	.257

*Note. N* = 409. *B *coefficients generated with model 4 of the PROCESS macro; *CI *confidence interval

### Outcome bias

Participants rated decisions with positive outcomes as better (*M* = 4.79, *SD* = 1.73) than those with negative outcomes (*M* = 3.04, *SD* = 1.57). This difference of 1.75, 95% CI [1.52, 1.98], was significant, *t*(408) = 14.81, *p* < .001, *d* = 0.73, 95% CI [0.62, 0.84]. We conducted a one-way ANOVA before grouping the two positive and the two negative versions. This analysis is in the supplemental material.

As there were differences between the versions with positive results, we were not able to group them and had to create dummy variables to conduct the analysis. The outcome bias model, *F*(8, 400) = 11.55, *p* < .001, *R*^2^ = .19, Table 3, indicated that conformity had a statistically significant negative direct effect, which means that the greater the conformity, the smaller the outcome bias. We did not identify other statistically significant direct effects, indirect effects, and interactions.

**Table 3:** Mediation model of the outcome bias

Variable	*B*	*SE*	95% CI for *B*	*p*
Self-direction	0.11	0.11	[−0.12, 0.33]	.349
Conformity	−0.26	0.11	[−0.48, −0.05]	.017
Power	−0.02	0.11	[−0.23, 0.19]	.191
Universalism	0.23	0.12	[−0.01, 0.48]	.059
Constant	1.07	0.21	[0.65, 1.49]	<.001
Need For Cognition	−0.17	0.11	[−0.38, 0.05]	.131
Version 2 ^a^	1.44	0.31	[0.83, 2.05]	<.001
Version 3 ^b^	1.81	0.31	[1.20, 2.42]	<.001
Version 4 ^c^	−0.63	0.30	[−1.22, −0.05]	.034

*Note. N* = 409*. B* coefficients generated with model 4 of the PROCESS macro; *CI* confidence interval

^a^Scenario in which the first stimulus presented a decision by a mayor with a negative result

^b^Scenario in which the first stimulus presented a decision by an investor with a positive result

^c^Scenario in which the first stimulus presented a decision by an investor with a negative result

As we identified differences in the results of the four cognitive biases studied, we also tested the correlations between these biases. We did not identify any statistically significant correlation between them, as shown in Table 4. The R script we used and its output, including confidence intervals, are available on OSF (https://osf.io/twqsu).

**Table 4:** Descriptive statistics and correlations between cognitive biases and individual differences

Variable	*n*	*M*	*SD*	1	2	3	4	5	6	7	8	9	10
1. Anchoring	363	26.67	58.63										
2. Framing	409	2.27	2.99	−.03									
3. Certainty	409	0.89	2.53	−.09	.03								
4. Outcome	409	1.75	2.39	−.02	−.05	−.08							
5. Self-direction	409	5.08	0.61	.12*	−.05	.07	.06						
6. Conformity	409	4.49	0.82	.01	.06	−.00	−.05	.05					
7. Power	409	2.76	1.08	.04	.02	−.12*	.01	.01	−.01				
8. Universalism	409	5.17	0.57	−.03	.06	−.01	.05	.31**	.34**	−.11**			
9. Need for Cognition	409	3.58	0.70	−.10	.04	.03	−.03	.23**	−.10*	−.09	.02		
10. Objective Numeracy	409	0.67	0.36	−.16**	.19**	.07	−.05	.03	−.05	.15**	−.10	.28**	
11. Subjective Numeracy	409	4.28	1.07	−.11*	.11**	.09	−.08	.01	−.05	.08	−.11*	.39**	.53**

*p < .05. **p < .01

## Discussion

This study examined the relationships between human values and cognitive biases, as well as the potential mediating effects of the need for cognition and numeracy moderation. The anchoring effect, framing effect, certainty effect, and outcome bias have consistently influenced initial choices and changes in preference, providing further evidence on the effects observed in earlier studies (e.g., Baron & Hershey, 1988; Larrick & Soll, 2008; Quattrone & Tversky, 1988; Tversky & Kahneman, 1974, 1981). However, the relationships of these biases with self-direction, conformity, power, universalism, the need for cognition, and numeracy were different for each of them, indicating the potential involvement of distinct psychological mechanisms. Previous studies have already shown that self-generated and provided anchors involve different adjustment processes (Epley & Gilovich, 2006; Simmons et al., 2010; Tversky & Kahneman, 1974). We identified a lack of correlations between the biases studied, which reinforces that there are several psychological processes involved. People more susceptible to one bias were not similarly susceptible to another. This finding may help both investigations that seek to improve human reasoning in situations where cognitive heuristics and biases are not suitable (Berthet, 2022; Bystranowski et al., 2021; Kahneman, 2011; Neal et al., 2022; Stanovich, 2016), as well as investigations that seek to obtain better performance where they are appropriate (Marewski & Gigerenzer, 2012; Mousavi & Gigerenzer, 2017). Although cognitive biases have been extensively researched, there still seems to be a need for further study of the mechanisms behind them (Berthet & De Gardelle, 2023).

Self-direction and its mediation by need for cognition were related only to the anchoring effect. As we expected the greater the self-direction, the lower the anchoring; however, the need for cognition relation was different from the study of Epley and Gilovich (2006), indicating that the greater need for cognition, the greater the anchoring. One possibility is that people who like to be more involved in understanding problems may have realized the intent of the stimuli and resisted adjusting their initial estimates. This was the only bias where we expected a greater preference maintenance effect than a preference change. In this sense, it is important to highlight that pre-registration was essential to record clearly and transparently, before data collection, that we would consider the self-generated anchor, instead of the most provided anchor approach (Tversky & Kahneman, 1974) in the regression analyses. Power was directly related only to the certainty effect. However, a greater appreciation of power was associated with a smaller change in preference. Power is associated with the concern with the control and domination of people and resources (Schwartz, 1992). The problems used to measure the certainty effect were the only ones involving preferences over the financial gains of the participants themselves. So, they may have paid more attention to these problems. Conformity was related only to outcome bias. While we expected that valuing subordination would be related to greater effects, an important point is that conformity can also motivate people to prefer to keep their opinions rather than openly disagree with others (Schwartz, 2012). Thus, greater conformity may be related to a lower degree of review of decisions taken, regardless of their results. Moderation by numeracy occurred only in problems related to the framing effect and certainty effect. In these problems, the normative decision depended on a greater degree of calculation, reinforcing the role of this apparatus in these choices. On the other hand, there was no moderation by numeracy in the outcome bias, which did not even use numbers, and in the anchoring effect. Future research may attempt to replicate the results found in this study, analyzing one or more of the investigated cognitive biases, and exploring other measures for these biases.

### Limitations and future directions

We cannot fail to mention some limitations of this research. Due to concerns about the size of the questionnaire, we did not explore all the values of Schwartz’s (1992) theory, focusing only on those that we thought would be related to the biases studied. Likewise, relationships with other cognitive biases, such as denominator neglect (Kahneman, 2011), can also be studied. Another limitation is that we did not investigate the relationship between the variables studied with the gender and age of the participants. Gender and age differences can influence human values (Borg. 2019; Leijen et al., 2022; Schwartz & Rubel, 2005). So, future studies can explore whether gender and age moderate associations between cognitive biases and values. As we mentioned, new studies can explore other measures for the studied biases and examine whether the lack of correlation between them is replicated. Furthermore, future research can investigate samples from countries other than Brazil for cross-cultural analysis. It is worth mentioning that, despite not being so common in cognitive bias studies, the use of within-participant stimuli was feasible. However, the issue we had with the two stimuli related to positive outcomes shows that the within-participant stimuli require great care in their elaboration. This study focused on the relationship of human values with cognitive bias rather than on models to explain all the variations in the bias effects. As with many mediation, moderation, and conditional process analyses, our interest was more in the regression coefficients than in the model’s overall fit (Hayes, 2018). Nonetheless, it is important to note that the low *R*^2^ reported values indicate that the models explain very little variance. Future studies aiming to investigate good-fitting models for the variances in bias effects should consider including other variables that might contribute to these variances.

## Conclusions

This study found new evidence of the effects of cognitive biases induced by the anchoring effect, the framing effect, the certainty effect, and the outcome bias, indicating the involvement of different psychological mechanisms. People more susceptible to one bias are not similarly susceptible to other biases. This is relevant to research on how to strengthen or weaken cognitive heuristics and biases. In addition, the differences observed, such as the association between the need for cognition and self-generated anchoring distinct from the study of Epley and Gilovich (2006), reinforce the difficulty in generalizing phenomena affected not only by how the stimuli are elaborated but also by the context in which they occur. In this sense, beyond the individual level, cross-cultural research can also investigate the relationship between cultural differences and bias effects (Kakinohana et al., 2023). Therefore, despite the extensive research already carried out on cognitive biases, there still appears to be a need for further study of the several mechanisms behind them.

## Abbreviations

ANOVA: Analysis of variance
EFS: Enterprise Feedback Suite
HC3: Heteroscedasticity consistent 3
IBM: International Business Machines
OSF: Open Science Framework
PVQ-RR: Revised Portrait Values Questionnaire
SPSS: Statistical Package for the Social Sciences

## References

[CR1] Baron J, Hershey JC (1988). Outcome bias in decision evaluation. Journal of Personality and Social Psychology.

[CR2] Berthet, V. (2022). The impact of cognitive biases on professionals’ decision-making: a review of four occupational areas. *Frontiers in Psychology, 12*, 802439. 10.3389/fpsyg.2021.802439 10.3389/fpsyg.2021.802439 PMC8763848 35058862

[CR3] Berthet V, De Gardelle V (2023). The heuristics-and-biases inventory: an open-source tool to explore individual differences in rationality. Frontiers in Psychology.

[CR4] Bojanowska A, Urbańska B (2021). Individual values and well-being: the moderating role of personality traits. International Journal of Psychology.

[CR5] Borg I (2019). Age- and gender-related differences in the structure and the meaning of personal values. Personality and Individual Differences.

[CR6] Bystranowski P, Janik B, Próchnicki M, Skórska P (2021). Anchoring effect in legal decision-making: a meta-analysis. Law and Human Behavior.

[CR7] Cacioppo JT, Petty RE (1982). The need for cognition. Journal of Personality and Social Psychology.

[CR8] Cacioppo JT, Petty RE, Feinstein JA, Jarvis WBG (1996). Dispositional differences in cognitive motivation: the life and times of individuals varying in need for cognition. Psychological Bulletin.

[CR9] Cacioppo JT, Petty RE, Kao CF (1984). The efficient assessment of need for cognition. Journal of Personality Assessment.

[CR10] Caldas LS, Iglesias F, Melo IR, Lyra RL (2019). Persuasion at different levels of elaboration: experimental effects of strength, valence and ego depletion. Trends in Psychology.

[CR11] Carnevale JJ, Inbar Y, Lerner JS (2011). Individual differences in need for cognition and decision-making competence among leaders. Personality and Individual Differences.

[CR12] Coelho GLH, Hanel PHP, Wolf LJ (2020). The very efficient assessment of need for cognition: developing a six-item version. Assessment.

[CR13] Cohen, J. (1988). *Statistical Power Analysis for the Behavioral Sciences* (2nd ed.). New York: Routledge.

[CR14] Cokely ET, Galesic M, Schulz E, Ghazal S, Garcia-Retamero R (2012). Measuring risk literacy: the Berlin Numeracy Test. Judgment and Decision Making.

[CR15] Epley N, Gilovich T (2001). Putting adjustment back in the anchoring and adjustment heuristic: differential processing of self-generated and experimenter-provided anchors. Psychological Science.

[CR16] Epley N, Gilovich T (2006). The anchoring-and-adjustment heuristic: why the adjustments are insufficient. Psychological Science.

[CR17] Evans J. St., B. T., & Stanovich, K. E.  (2013). Dual-process theories of higher cognition: advancing the debate. Perspectives on Psychological Science.

[CR18] Faul, F., Erdfelder, E., Buchner, A., & Lang, A.-G. (2009). Statistical power analyses using G*Power 3.1: tests for correlation and regression analyses. *Behavior Research Methods, 41*(4), 1149–1160. 10.3758/BRM.41.4.1149 10.3758/BRM.41.4.1149 19897823

[CR19] Fagerlin A, Zikmund-Fisher BJ, Ubel PA, Jankovic A, Derry HA, Smith DM (2007). Measuring numeracy without a math test: development of the Subjective Numeracy Scale. Medical Decision Making.

[CR20] Field, A. (2018). *Discovering statistics using IBM SPSS statistics* (5th ed.). London: Sage.

[CR21] Fischer, R., Bortolini, T., Pilati, R., Porto, J., & Moll, J. (2021). Values and COVID-19 worries: the importance of emotional stability traits. *Personality and Individual Differences, 182*, 111079. 10.1016/j.paid.2021.111079 10.1016/j.paid.2021.111079 PMC8439713 34538995

[CR22] Fleischhauer M, Enge S, Brocke B, Ullrich J, Strobel A, Strobel A (2010). Same or different? Clarifying the relationship of need for cognition to personality and intelligence. Personality and Social Psychology Bulletin.

[CR23] Furnham A, Thorne JD (2013). Need for cognition: its dimensionality and personality and intelligence correlates. Journal of Individual Differences.

[CR24] Ghazal S, Cokely ET, Garcia-Retamero R (2014). Predicting biases in very highly educated samples: numeracy and metacognition. Judgment and Decision Making.

[CR25] Goren P, Smith B, Motta M (2022). Human values and sophistication interaction theory. Political Behavior.

[CR26] Grosz MP, Schwartz SH, Lechner CM (2021). The longitudinal interplay between personal values and subjective well-being: a registered report. European Journal of Personality.

[CR27] Hayes, A. F. (2018). *Introduction to mediation, moderation, and conditional process analysis: a regression-based approach* (2nd ed.). New York: Guilford Press.

[CR28] Kahneman D (2011). Thinking fast and slow.

[CR29] Kahneman D, Tversky A (1984). Choices, values, and frames. American Psychologist.

[CR30] Kakinohana RK, Pilati R, Klein RA (2023). Does anchoring vary across cultures? Expanding the many labs analysis. European Journal of Social Psychology.

[CR31] Kharlamov A, Pogrebna G (2021). Using human values-based approach to understand cross-cultural commitment toward regulation and governance of cybersecurity. Regulation & Governance.

[CR32] Klein G, Ben Hador B (2021). Recruitment agencies and unethical client requests: the ‘loyal matchmaker’ dilemma. The Irish Journal of Management.

[CR33] Larrick RP, Soll JB (2008). The MPG illusion. Science.

[CR34] Leijen I, Van Herk H, Bardi A (2022). Individual and generational value change in an adult population, a 12-year longitudinal panel study. Scientific Reports.

[CR35] Liberali JM, Reyna VF, Furlan S, Stein LM, Pardo ST (2012). Individual differences in numeracy and cognitive reflection, with implications for biases and fallacies in probability judgment. Journal of Behavioral Decision Making.

[CR36] Lipkus IM, Samsa G, Rimer BK (2001). General performance on a numeracy scale among highly educated samples. Medical Decision Making.

[CR37] Long JS, Ervin LH (2000). Using heteroscedasticity consistent standard errors in the linear regression model. The American Statistician.

[CR38] Marewski, J. N., & Gigerenzer, G. (2012). Heuristic decision making in medicine. *Dialogues in clinical neuroscience*, *14*(1), 77–89. 10.31887/DCNS.2012.14.1/jmarewski 10.31887/DCNS.2012.14.1/jmarewski PMC3341653 22577307

[CR39] Mousavi S, Gigerenzer G (2017). Heuristics are tools for uncertainty. Homo Oeconomicus.

[CR40] Mubako G, Bagchi K, Udo G, Marinovic M (2021). Personal values and ethical behavior in accounting students. Journal of Business Ethics.

[CR41] Neal TMS, Lienert P, Denne E, Singh JP (2022). A general model of cognitive bias in human judgment and systematic review specific to forensic mental health. Law and Human Behavior.

[CR42] Peters E, Västfjäll D, Slovic P, Mertz CK, Mazzocco K, Dickert S (2006). Numeracy and decision making. Psychological Science.

[CR43] Quattrone GA, Tversky A (1988). Contrasting rational and psychological analyses of political choice. American Political Science Review.

[CR44] Reyna VF, Brainerd CJ (2023). Numeracy, gist, literal thinking and the value of nothing in decision making. Nature Reviews Psychology.

[CR45] Roberts PS, Wernstedt K (2019). Decision biases and heuristics among emergency managers: just like the public they manage for?. The American Review of Public Administration.

[CR46] Sagiv L, Schwartz SH (2022). Personal values across cultures. Annual Review of Psychology.

[CR47] Schwartz LM (1997). The role of numeracy in understanding the benefit of screening mammography. Annals of Internal Medicine.

[CR48] Schwartz, S. H. (1992). Universals in the content and structure of values: theoretical advances and empirical tests in 20 countries. In M. P. Zanna (Ed.), *Advances in Experimental Social Psychology* (Vol. 25, pp. 1–65). Academic Press. 10.1016/S0065-2601(08)60281-6

[CR49] Schwartz SH (2006). Les valeurs de base de la personne: Théorie, mesures et applications Basic human values: theory, measurement, and applications. Revue Française de Sociologie.

[CR50] Schwartz SH (2006). A theory of cultural value orientations: explication and applications. Comparative Sociology.

[CR51] Schwartz, S. H. (2012). An overview of the Schwartz Theory of Basic Values. *Online Readings in Psychology and Culture, 2*(1). 10.9707/2307-0919.1116

[CR52] Schwartz, S. H. (2016). *Coding and analyzing PVQ-RR data (instructions for the revised Portrait Values Questionnaire)*. 10.13140/RG.2.2.35393.56165

[CR53] Schwartz, S. H. (2017). The refined theory of basic values. In S. Roccas & L. Sagiv (Eds.), *Values and behavior: Taking a cross cultural perspective* (pp. 51–72). Springer International Publishing. 10.1007/978-3-319-56352-7_3

[CR54] Schwartz SH, Cieciuch J (2022). Measuring the refined theory of individual values in 49 cultural groups: psychometrics of the Revised Portrait Value Questionnaire. Assessment.

[CR55] Schwartz SH, Cieciuch J, Vecchione M, Davidov E, Fischer R, Beierlein C, Ramos A, Verkasalo M, Lönnqvist J-E, Demirutku K, Dirilen-Gumus O, Konty M (2012). Refining the theory of basic individual values. Journal of Personality and Social Psychology.

[CR56] Schwartz SH, Rubel T (2005). Sex differences in value priorities: cross-cultural and multimethod studies. Journal of Personality and Social Psychology.

[CR57] Simmons JP, LeBoeuf RA, Nelson LD (2010). The effect of accuracy motivation on anchoring and adjustment: do people adjust from provided anchors?. Journal of Personality and Social Psychology.

[CR58] Simon HA (1955). A behavioral model of rational choice. Quarterly Journal of Economics.

[CR59] Šrol J, De Neys W (2021). Predicting individual differences in conflict detection and bias susceptibility during reasoning. Thinking & Reasoning.

[CR60] Stanovich KE (2016). The comprehensive assessment of rational thinking. Educational Psychologist.

[CR61] Stanovich KE (2018). Miserliness in human cognition: the interaction of detection, override and mindware. Thinking & Reasoning.

[CR62] Stanovich KE, West RF (2008). On the relative independence of thinking biases and cognitive ability. Journal of Personality and Social Psychology.

[CR63] Tabachnick, B. G., & Fidell, L. S. (2018). *Using multivariate statistics* (7th ed.). New York: Pearson.

[CR64] Tamayo, A., & Porto, J. B. (2009). Validação do Questionário de Perfis de Valores (QPV) no Brasil [Validity of the Portrait Values Questionnaire (PVQ) in Brazil]. *Psicologia: Teoria e Pesquisa, 25*(3), 369–376. 10.1590/S0102-37722009000300010

[CR65] Thaler RH, Sunstein CR (2009). Nudge: improving decisions about health, wealth, and happiness.

[CR66] Thomson KS, Oppenheimer DM (2016). Investigating an alternate form of the cognitive reflection test. Judgment and Decision Making.

[CR67] Toplak ME, Flora DB (2021). Resistance to cognitive biases: longitudinal trajectories and associations with cognitive abilities and academic achievement across development. Journal of Behavioral Decision Making.

[CR68] Toplak ME, West RF, Stanovich KE (2014). Rational thinking and cognitive sophistication: development, cognitive abilities, and thinking dispositions. Developmental Psychology.

[CR69] Torres CV, Schwartz SH, Nascimento TG (2016). A Teoria de Valores Refinada: Associações com comportamento e evidências de validade discriminante e preditiva [The Refined Value Theory: associations with behaviour and evidence of discriminant and predictive validity]. Psicologia USP.

[CR70] Tversky A, Kahneman D (1974). Judgment under uncertainty: heuristics and biases. Science.

[CR71] Tversky A, Kahneman D (1981). The framing of decisions and the psychology of choice. Science.

[CR72] West RF, Toplak ME, Stanovich KE (2008). Heuristics and biases as measures of critical thinking: associations with cognitive ability and thinking dispositions. Journal of Educational Psychology.

[CR73] Wyszynski M, Diederich A (2023). Individual differences moderate effects in an unusual disease paradigm: a psychophysical data collection lab approach and an online experiment. Frontiers in Psychology.

[CR74] Yamagishi, K. (1997). When a 12.86% mortality is more dangerous than 24.14%: implications for risk communication. *Applied Cognitive Psychology,**11*(6), 495–506. 10.1002/(SICI)1099-0720(199712)11:63.0.CO;2-J

